# Supplementary material for: The impact of internet use on the subjective well-being of older adults: The mediating role of mental health
Source: PLoS One. 2025 Oct 31;20(10):e0335985. doi: 10.1371/journal.pone.0335985 (PMC12578345; doi:10.1371/journal.pone.0335985)
Supplement: Attachment — Submitted filename: Response to Reviewers.docx [file pone.0335985.s002.docx]

**Response to Academic Editor and Reviewers**

**Manuscript Title: *The Impact of Internet Use on the Subjective Well-Being of Older Adults: The Mediating Role of Mental Health***

**Manuscript Number: [PONE-D-24-57555]**

Dear Academic Editor and Reviewers,

On behalf of all co-authors, we would like to express our sincere gratitude for your thoughtful and constructive comments on our manuscript. We greatly appreciate the time and effort you have invested in reviewing our work. Your feedback has significantly improved the clarity, rigor, and academic contribution of the paper.

Based on your suggestions, we have made careful and substantial revisions throughout the manuscript. All changes have been marked using the Track Changes function for your review. Below, we provide a point-by-point response to each of the editor’s and reviewers’ comments. Original comments are presented in black, and our responses are in red for clarity.

We sincerely hope that the revised version meets your expectations, and we once again thank you for your valuable time and effort.

**Response to Academic Editor’s Comments：**

**New Editorial Comments 1:** Please note that your Data Availability Statement is currently missing the DOI/accession number of each dataset OR a direct link to access each database. If your manuscript is accepted for publication, you will be asked to provide these details on a very short timeline. We therefore suggest that you provide this information now, though we will not hold up the peer review process if you are unable.

**Response 1:** We thank the editorial office for highlighting this important requirement regarding data accessibility. The data used in our study were obtained from the 2021 wave of the Chinese General Social Survey (CGSS), hosted by the National Survey Research Center at Renmin University of China. The dataset is accessible through the Chinese National Survey Data Archive (CNSDA). We have now updated the Data Availability Statement both at the end of the manuscript and in a separate supplementary file to include the direct access link to the dataset, as follows:

http://www.cnsda.org/index.php?r=projects/view&id=65635422

This link leads to the official data application page, where users may register, describe their research project, and submit a request for access. As stated, the authors did not receive any special privileges during data acquisition. We hope this revision meets the journal’s expectations for transparency and data availability. Please feel free to let us know if any further clarification is required.

**New Editorial Comments 2:** Please ensure that you refer to Figure 1 in your text as, if accepted, production will need this reference to link the reader to the figure.

**Response 2:** We appreciate this helpful reminder. In response, we have added a direct reference to Figure 1 in the section titled “Research Objectives and Hypotheses”, specifically in line 144 of the revised manuscript. The newly added sentence reads:

“Figure 1 presents the proposed structural model, which guides the formulation of the research hypotheses outlined below.”

This change ensures that Figure 1 is properly linked to the main text and can be correctly processed during production. Please let us know if any further clarification is needed.

**Comments 1:** When submitting your revision, we need you to address these additional requirements. Please ensure that your manuscript meets PLOS ONE's style requirements, including those for file naming. The PLOS ONE style templates can be found at https://journals.plos.org/plosone/s/file?id=wjVg/PLOSOne_formatting_sample_main_body.pdf and https://journals.plos.org/plosone/s/file?id=ba62/PLOSOne_formatting_sample_title_authors_affiliations.pdf

**Response 1:** We appreciate your kind reminder regarding PLOS ONE’s formatting standards. In response, we have thoroughly reviewed and implemented the journal’s style requirements. Specifically, we have:

1. Formatted the main manuscript according to the PLOS ONE main body template, including proper section headings, figure/table callouts, and structure.
2. Ensured that the title page and author affiliation section are presented in accordance with the designated template.
3. Updated the file naming conventions to comply with PLOS ONE’s submission policies (e.g., “Manuscript.docx” was renamed accordingly before upload).

We hope these revisions meet the journal’s formatting expectations. Please kindly let us know if any further adjustments are needed.

**Comments 2:** Please provide a complete Data Availability Statement in the submission form, ensuring you include all necessary access information or a reason for why you are unable to make your data freely accessible. If your research concerns only data provided within your submission, please write "All data are in the manuscript and/or supporting information files" as your Data Availability Statement.

**Response 2:** Thank you for your comment on the Data Availability Statement. We confirm that the data used in this study come from the 2021 Chinese General Social Survey (CGSS), which is managed by the National Survey Research Center (NSRC) at Renmin University of China. As the data are not publicly downloadable, researchers must apply for access via the CGSS official website (http://cgss.ruc.edu.cn/), and no special access privileges were granted to the authors.

We have clarified this information in the Data Availability Statement section of our manuscript and ensured that it aligns with PLOS ONE’s data sharing policy.

**Comments 3:** First, since your study focuses on the mediating roles of mental health-related variables: psychological anxiety, social loneliness, and goal deficiency in the relationship between internet use and well-being, it would be beneficial to elaborate on the mediating effects of each variable rather than discussing mental health in general. As Reviewer 2 pointed out, the literature review needs to be significantly strengthened. Additionally, the rationale for conceptualizing mental health as encompassing these three specific dimensions should be well-justified with theoretical support.

**Response 3:** We appreciate your valuable suggestions. The manuscript has been revised as follows to address your points: Expanded Mediation Analysis: In both the Results and Discussion sections, we have separately elaborated on the mediating effects of each psychological variable—psychological anxiety, social loneliness, and goal deficiency—rather than treating mental health as a single general construct. For example, we highlighted that the path via psychological anxiety showed the strongest indirect effect, while the effect of goal deficiency was weaker but still significant, and the effect of social loneliness was not statistically significant.

1. Enhanced Literature Review: In the revised Literature Review section, we have substantially expanded the discussion to include recent (post-2020) studies on internet use and mental health among older adults. These references better reflect the current state of the field and provide stronger empirical foundations for our research hypotheses (H1–H5).
2. Theoretical Justification: We have added a new paragraph to explain the theoretical basis for selecting the three psychological dimensions. Specifically: Psychological anxiety is grounded in the stress-coping theory; Social loneliness draws on social support theory; Goal deficiency is supported by motivational theory and meaning-making frameworks.

This addition appears in both the Literature Review and Method sections (specifically under the “Mediating Variables” subsection), with a clear operationalization of each construct based on validated items from the CGSS dataset.

We hope these revisions provide a clearer and more rigorous foundation for our conceptual model and empirical analyses.

**Comments 4:** Second, Tables 2–5 consistently show positive relationships among key variables. For example, in Table 3, the coefficient of 0.193 indicates that higher psychological anxiety was positively associated with subjective well-being—an unexpected finding that contradicts existing literature. If taken at face value, this result would suggest that increasing older adults’ anxiety levels could enhance their well-being, which is counterintuitive. Additionally, the internet use was positively associated with three mediators (i.e., psychological anxiety, social loneliness, and goal deficiency) as shown in Table 4. However, the discussion on page 23 interprets the findings contradictorily: “This finding aligns with the perspective of social compensation theory [36], suggesting that social media plays an especially prominent role in alleviating psychological anxiety.” Similarly, although goal deficiency was found to be positively related to subjective well-being in Table 3, the discussion on page 22 states: “Similarly, the reduction in goal deficiency also significantly mediated the relationship between social media use and subjective well-being. Social media provides crucial support for older adults in rebuilding their sense of purpose by offering opportunities to participate in community activities, express themselves, and learn new skills.” These interpretations seem inconsistent with the reported results. Please carefully review your data analysis procedures and ensure that the findings are precise and accurately reflected in the discussion.

**Response 4:** We greatly appreciate your careful attention to the consistency between the empirical results and theoretical interpretations. We sincerely apologize for the earlier inconsistency between the regression findings and the theoretical discussion, especially regarding psychological anxiety and goal deficiency. After carefully reviewing the regression results and indirect effect pathways, we have revised the relevant sections in the Discussion and Results parts to ensure consistency and accuracy in interpretation:

1. Psychological Anxiety (Table 3): The unstandardized coefficient of psychological anxiety in Table 3 is –0.193, not +0.193. We reviewed the manuscript and confirmed that this may have resulted from a typographical oversight in the previous draft. The correct interpretation — that higher anxiety is associated with lower subjective well-being — is now consistently reflected in the discussion. We have added clarification in the first and second paragraphs of the discussion to align with both Table 3 and the indirect effect shown in Table 4.
2. Goal Deficiency: Table 3 also shows a negative relationship between goal deficiency and subjective well-being. The mediation effect presented in Table 4 further supports this indirect pathway. In the earlier version, we did not fully elaborate on the mechanism. We have now refined our explanation to clearly state that a reduction in goal deficiency contributes to improved well-being, and we have revised this in the third paragraph of the discussion section.
3. Social Loneliness: While social loneliness was not a significant mediator in the indirect path model, we acknowledge that the previous discussion may have overstated its impact. We have revised the wording to emphasize that its mediating effect was not statistically significant, consistent with Table 4. This correction is included in the third paragraph of the revised discussion.
4. Theoretical Framing: To enhance conceptual clarity and ensure theoretical alignment, we have reinforced the interpretation using flow theory and social compensation theory. The causal logic linking internet use, psychological mediators, and subjective well-being is now clearly stated. All revised interpretations are now directly grounded in the empirical findings from Tables 3 and 4.

**Comments 5:** Furthermore, regarding Table 5, the direct effect between internet use and subjective well-being is reported as 0.025, yet the total effect (direct + indirect) is the same as the total indirect effect (0.024). Please verify these statistics. Last, please report the procedures of getting the indirect effects contrast in Table 5.

**Response 5:** We appreciate your close scrutiny of the reported model estimates and your attention to statistical detail. We sincerely apologize for the confusion caused by the inconsistent effect values reported in the previous version. In response, we have taken the following corrective actions:

1. We re-estimated the structural equation model using the R lavaan package, which allowed us to compute all path coefficients and mediation effects within a consistent framework. The previous inconsistency was due to a typographical oversight during table construction, which has now been corrected.
2. The revised Table 4 now presents standardized estimates for the direct, indirect, and total effects, as well as z-values, p-values, and 95% confidence intervals. The total effect of Internet Use on Subjective Well-being is now correctly reported as 0.066, which matches the sum of the direct effect (0.059) and the total indirect effect (0.029). This correction ensures statistical coherence and transparency.
3. Regarding the procedure for estimating indirect effects, we used bootstrapping with 5,000 samples to calculate bias-corrected confidence intervals for each indirect pathway. This approach is implemented via the sem() function and parameterEstimates() output in the lavaan package, which directly provides the standardized estimates, z-values, p-values, and confidence intervals used in Table 4.

We hope this resolves the issue and greatly appreciate your careful reading and valuable feedback.

**Response to Reviewer 1’s Comments:**

**Comments 1:** Statistical analyses were performed using SPSS and the PROCESS macro. I suggest using path analysis in software such as R, LISREL, or AMOS. In this way, fit indices, which are very important in modeling, are also reported.

**Response 1:** Building upon your insightful suggestion, we have implemented structural equation modeling (SEM) using the lavaan package in R to enhance analytical robustness and clarity. This approach enabled us to simultaneously estimate direct, indirect, and total effects, and importantly, to include model fit indices.

In the revised version of our manuscript, we have re-conducted the mediation analysis using the lavaan package in R. The SEM approach enabled us to simultaneously estimate direct, indirect, and total effects, and more importantly, to report a set of model fit indices. As a result, we have:

1. Reconstructed Table 4 to present standardized path coefficients, z-values, p-values, and confidence intervals for all key paths.
2. Added Table 5, which reports model fit indices including χ²/df, RMSEA, GFI, AGFI, NFI, NNFI, and IFI, all of which indicate acceptable model fit according to conventional criteria.

We believe that this change has greatly improved the transparency and methodological rigor of our analysis. The corresponding descriptions in the Results section have also been updated accordingly.

**Comments 2:** Assumptions of statistical analysis should be checked and reported.

**Response 2:** We fully recognize the importance of verifying and transparently reporting the assumptions underlying statistical analysis. In response to your suggestion, we carefully re-examined our analytical procedures and have added explicit clarification in the Statistical Analysis subsection of the Methods section. Specifically, we now state that essential assumptions—such as normality, linearity, and multicollinearity—were assessed prior to conducting the structural equation modeling (SEM) using the lavaan package in R. Additionally, model fit indices (e.g., RMSEA, GFI, NFI, CFI) are now reported in the newly added Table 5, in alignment with conventional SEM reporting standards. We sincerely hope that these improvements address your concern and enhance the methodological rigor and transparency of the study. Thank you again for your constructive feedback.

**Comments 3:** Another advantage of using path analysis in the way I mentioned is that all hypotheses are tested simultaneously, and a path diagram is also reported. Your results are fragmented.

**Response 3:** We fully concur with your observation that structural path modeling provides a more integrated and interpretable framework for testing all hypothesized relationships simultaneously. We also agree that the inclusion of a path diagram can significantly improve the clarity of empirical findings. Following your helpful suggestion, we replaced the initial stepwise regression analysis with a structural equation model (SEM) using the lavaan package in R. This allowed us to examine the direct and indirect pathways from internet use to subjective well-being through psychological anxiety, social loneliness, and goal deficiency within a unified model. The updated results are now summarized in Table 4, and we have also added a path diagram (Figure 2) that visually illustrates the standardized coefficients and significance levels of each path. We are grateful for your recommendation, which has helped us enhance both the analytical rigor and the presentation quality of our findings.

**Comments 4:** There is an error in reporting one of the sources on line 411. Please correct it.

**Response 4:** We appreciate your careful attention to detail regarding the citation error on line 411. We sincerely apologize for this oversight. Upon careful review, we found that the reference was either misreported or improperly formatted in the previous version of the manuscript. We have now corrected the citation accordingly to ensure its accuracy and consistency with the formatting requirements. We have also re-checked all references throughout the manuscript to ensure there are no similar issues. We are grateful for your attention to detail and constructive feedback, which has helped improve the overall quality of the manuscript.

**Response to Reviewer 2’s Comments:**

**Comments 1:** Literature review: the literature review is too simplistic and descriptive. It needs to be enhanced and enriched by better linking with the research hypotheses.

**Response 1:** We acknowledge the reviewer’s insightful observation regarding the simplicity and descriptive nature of the original literature review. In response, we have substantially revised this section to improve its conceptual depth, theoretical alignment, and connection to our hypotheses. The following key enhancements were made:

1. Strengthened Theoretical Integration: We revised the literature review to incorporate a clearer conceptual framework linking internet use with subjective well-being through psychological mediators. Specifically, we introduced and elaborated on Flow Theory, Social Compensation Theory, Self-Determination Theory, and Activity Theory to explain the hypothesized mechanisms. These theories now serve as a foundation for our hypotheses development and are explicitly referenced in the revised manuscript (see “Introduction” and “Literature Review” sections).
2. Detailed Expansion of Key Constructs: We expanded the descriptions of all core concepts, including psychological anxiety, social loneliness, and goal deficiency, grounding them in representative studies (e.g., Deci & Ryan, 2000; Ryff & Keyes, 1995) and emphasizing how they influence well-being among older adults. We also discussed how each construct has been operationalized in previous empirical research and justified their inclusion as mediators in this study.
3. Clearer Link to Research Hypotheses: We restructured the final part of the literature review to more directly connect empirical findings and theoretical insights to our five research hypotheses (H1–H5). Each paragraph now ends with a hypothesis statement supported by both theoretical and empirical justifications.

**Comments 2:** Sample Representativeness: The sample primarily consists of older adults from rural areas, which may not fully capture the characteristics of internet use among urban older adults. The generalizability of the findings to diverse populations could be questioned.

**Response 2:** Thank you very much for this important observation. We fully acknowledge the limitation raised regarding the representativeness of our sample. The data used in this study were drawn from the 2021 wave of the Chinese General Social Survey (CGSS), which employed stratified multi-stage sampling across various regions. However, due to data availability and the specific filtering criteria applied (e.g., complete responses on key variables), our final analytic sample indeed includes a disproportionately high percentage of older adults from rural areas. In response to your suggestion, we have made the following updates in the manuscript:

1. Added a limitation discussion: In the Discussion section, we have explicitly acknowledged this rural sampling bias and noted that older adults in urban settings may display different patterns of internet use and digital engagement.
2. Clarified implications for generalizability: We added a remark that the generalizability of the findings should be interpreted with caution, and we encouraged future research to adopt stratified or regionally balanced sampling designs to validate and extend our findings.

**Comments 3:** Measurement of Internet Use: The measurement of internet use is based on a single question about frequency, which may not capture the complexity of internet usage patterns (e.g., active vs. passive use, types of activities). A more nuanced measurement could provide deeper insights into the mechanisms at play.

**Response 3:** We recognize the validity of your observation concerning the limitations of using a single-item frequency measure to capture internet use. As you rightly pointed out, relying solely on usage frequency overlooks the complexity and diversity inherent in digital behaviors—particularly among older adults, whose online activities can differ significantly in intention, content, and engagement style. Our study utilizes secondary data from the 2021 Chinese General Social Survey (CGSS), which, due to its large-scale national sampling and standardization, only provides a single-item indicator of internet use frequency. While this variable has been widely adopted in prior research for population-level analysis, we acknowledge that it lacks the granularity needed to distinguish between active vs. passive usage, social vs. informational activities, or instrumental vs. recreational engagement. To address your important concern, we have made the following revisions in the Discussion section of the manuscript:

1. We now explicitly acknowledge this limitation and its implications for internal validity and explanatory power.
2. We suggest that future studies should employ multi-item and behaviorally specific instruments—including scales that distinguish purpose, content, and intensity of internet use—especially when analyzing psychological mechanisms and individual-level outcomes.

We appreciate your suggestion, which has helped us to critically reflect on the constraints of our data and offer clearer direction for future research.

**Comments 4:** Mediating Variables: While the study focuses on psychological anxiety, social loneliness, and goal deficiency as mediating variables, other potential mental health factors (e.g., depression, self-efficacy) could also play significant roles and should be considered in future research.

**Response 4:** We appreciate your thoughtful observation regarding the broader spectrum of psychological constructs relevant to older adults’ well-being. Indeed, variables such as depression, self-efficacy, and perceived control are well-established in the literature as important mediators in digital health research. In this study, our selection of psychological anxiety, social loneliness, and goal deficiency as mediators was guided by both data availability in the 2021 CGSS and established theoretical frameworks. Specifically, these three dimensions map onto key facets of emotional, social, and existential well-being, and align with theories such as stress-coping theory, social support theory, and goal-orientation theory, which we elaborated upon in the revised literature review and methodology sections. To address your concern, we have revised the Discussion section to acknowledge the limitations of our current mediating model and explicitly suggest the inclusion of additional psychological variables such as depressive symptoms, digital self-efficacy, and social anxiety in future studies. These additions would provide a more comprehensive understanding of the psychosocial pathways through which digital engagement influences well-being. Once again, thank you for this valuable recommendation, which has helped us to clarify the scope of our model and identify important directions for further research.

**Comments 5:** Discussion of Non-Significant Findings: The non-significant mediating effect of social loneliness should be discussed in greater depth, considering alternative explanations and implications for future research.

**Response 5:** We sincerely appreciate your insightful suggestion regarding the non-significant mediating role of social loneliness. You are right that our original manuscript did not sufficiently address this issue. In the revised Discussion section (paragraph 4), we have expanded the interpretation to offer a more detailed and context-sensitive explanation. While social loneliness is conceptually important, its mediating effect in this study was not statistically significant, which may be attributed to several factors:

1. Quality vs. quantity of online interaction: Social media may increase the frequency of online communication, but does not necessarily fulfill the emotional depth required to alleviate loneliness—especially among older adults, who often seek meaningful interpersonal relationships rather than superficial contacts.
2. Digital literacy disparities: A significant portion of our sample consists of older adults from rural areas, who may face limitations in digital skills, access, or platform familiarity. This could reduce the likelihood of engaging in emotionally rewarding online interactions.
3. Measurement limitations: Our study used a single-item self-report to assess loneliness, which may not capture the multidimensional nature of social loneliness (e.g., emotional vs. social loneliness).

In light of these factors, we have now emphasized the need for future studies to (1) use more comprehensive and validated tools to measure loneliness, (2) consider qualitative aspects of online engagement, and (3) explore moderating factors such as platform type or communication depth. We sincerely thank the reviewer for encouraging us to strengthen this important part of the discussion.

**Comments 6:** Policy Recommendations: The policy recommendations could be more specific, outlining concrete steps or examples of interventions that could be implemented based on the study's findings.

**Response 6:** Thank you very much for your thoughtful comment regarding the specificity of the policy recommendations. In the revised Conclusion section (paragraphs 2–3), we have substantially enhanced the policy implications by incorporating clear, actionable suggestions directly derived from our findings:

1. Digital literacy training: We recommend launching targeted training programs to improve older adults’ ability to use digital platforms actively and meaningfully, especially in rural areas where digital engagement is limited.
2. Platform optimization: Based on the finding that psychological anxiety is a key mediating variable, social media platforms could be optimized to include features that support emotional expression, stress relief, and community-building (e.g., peer forums, mental health chatbot support, guided interaction templates).
3. Mental health integration: We suggest integrating mental health services into digital environments, such as embedding virtual counseling, social companionship programs, or mindfulness content into applications frequently used by older adults.
4. Inclusive design policies: Policymakers should support the development of age-friendly interfaces, incorporating accessibility standards and culturally appropriate content to improve emotional connection and sense of purpose online.

We sincerely thank the reviewer for prompting us to improve the clarity and relevance of our recommendations. These enhancements aim to ensure our study not only contributes academically but also offers practical value to policymakers and social service providers.

**Comments 7:** Language and Clarity: Some sections of the manuscript could benefit from more concise and clear language to improve readability. For example, the abstract and introduction could be streamlined to highlight key points more effectively.

**Response 7:** We appreciate your insightful recommendation concerning language clarity and the organization of key sections. In response, we have substantially revised both the Abstract and the Introduction to improve clarity, conciseness, and readability. The abstract now follows a more structured format that clearly presents the background, methods, key findings, and implications. In the introduction, we have removed redundant content, clarified the research purpose, and streamlined the flow to better highlight the research gap and relevance. We also conducted a full language polish throughout the manuscript to enhance fluency and eliminate ambiguity. These changes have significantly improved the overall quality and readability of the paper. All revisions are marked in the track-changed version.

**Comments 8:** Data Presentation: The presentation of descriptive statistics and correlation results could be enhanced with more visual aids (e.g., tables, figures) to facilitate easier interpretation.

**Response 8:** We greatly appreciate your thoughtful suggestion regarding the visual presentation of empirical results. In response, we have taken several steps to enhance both the clarity and accessibility of key statistical findings:

1. Addition of Figure 2: We have added a new figure (Figure 2) that presents a standardized path diagram including all direct and indirect paths, along with corresponding t-statistics. This visualization helps clarify the structural relationships tested in the SEM model and enhances readers’ understanding of the mediating pathways.
2. Restructuring of Tables: Table 4 has been revised to clearly display the standardized coefficients, z-values, p-values, and 95% confidence intervals for all direct, indirect, and total effects. Table 5 now includes a comprehensive set of model fit indices (e.g., RMSEA, GFI, NFI, IFI) to assess the adequacy of the SEM model.
3. Improved Clarity and Layout: Both figure and tables have been formatted for readability and compliance with journal formatting guidelines.

We sincerely hope these enhancements improve the overall presentation and interpretation of our empirical findings.

Once again, we would like to express our heartfelt gratitude to the academic editor and both reviewers for your careful evaluation and constructive feedback. Your suggestions have significantly improved the clarity, methodological rigor, and theoretical contribution of our manuscript. We have done our best to address all comments with corresponding revisions and explanations.

We sincerely hope that the revised manuscript meets your expectations and is now suitable for publication in PLOS ONE. Please do not hesitate to let us know if any further revisions or clarifications are needed. We greatly appreciate your time and support.

With kind regards,

Chunyun Tan

Corresponding Author (on behalf of all authors)
