# Supplementary material for: The impact of internet use on the subjective well-being of older adults: The mediating role of mental health
Source: PLoS One. 2025 Oct 31;20(10):e0335985. doi: 10.1371/journal.pone.0335985 (PMC12578345; doi:10.1371/journal.pone.0335985)
Supplement: Attachment — Submitted filename: Response_to_Reviewers_auresp_2.docx [file pone.0335985.s003.docx]

**Response to Academic Editor and Reviewers**

**Manuscript Title: *The Impact of Internet Use on the Subjective Well-Being of Older Adults: The Mediating Role of Mental Health***

**Manuscript Number: [PONE-D-24-57555]**

Dear Academic Editor and Reviewers,

We would like to express our sincere gratitude to the academic editor and all reviewers for your thorough evaluation and constructive feedback on our manuscript. We greatly appreciate the time and effort you invested in helping us improve the clarity, conceptual grounding, and methodological rigor of this study.

In response to each comment, we have carefully revised the manuscript and provided point-by-point answers below. For clarity, each reviewer comment is restated, followed by our detailed response and the specific location of the corresponding changes in the revised manuscript.

**Response to Reviewer 3’s Comments:**

**Comments 1:** In the first paragraph of the introduction, you stated: “Against this backdrop, the widespread adoption of the internet is gradually becoming a key pathway to enhancing the subjective well-being of older adults. Exploring its underlying mechanisms holds significant theoretical value and practical implications.” (lines 46–49). This statement appears somewhat arbitrary, as the existing literature provides contradictory evidence on whether internet use improves or reduces users’ well-being. Your study focuses solely on the positive effects of internet use on subjective well-being. Please provide stronger justification for your research aim and situate it within the broader context of internet use literature. Referring to works such as Çikrıkci (2016) and Heo et al. (2015) may help strengthen your argument:

Çikrıkci, Ö. (2016). The effect of internet use on well-being: Meta-analysis. Computers in Human Behavior, 65, 560–566. https://doi.org/10.1016/j.chb.2016.09.021

Heo, J., Chun, S., Lee, S., Lee, K. H., & Kim, J. (2015). Internet use and well-being in older adults. Cyberpsychology, Behavior, and Social Networking, 18(5), 268–272.

**Response 1:** Thank you for this valuable comment. We agree that the original statement in the introduction could benefit from a more nuanced and balanced perspective. In the revised manuscript，we have incorporated a broader view of the literature, including studies that report both positive and more critical findings on the relationship between internet use and subjective well-being. Specifically, we have referred to Çikrıkci (2016) and Heo et al. (2015), whose work underscores the complexity and variability of this relationship. These revisions aim to provide stronger justification for our research aim and highlight the theoretical relevance of investigating the underlying mechanisms through which internet use may affect older adults’ well-being.

Manuscript revision location: Introduction, lines 52–57.

**Comment 2:** The third paragraph of the introduction attempts to justify why you focus on mental health as a potential mechanism underlying the relationship between internet use and subjective well-being. However, the current arguments, such as moderate or excessive internet use and the social compensation theory, focus more on how people use the internet and which kinds of people benefit in terms of well-being. These points address the boundary conditions of the relationship between internet use and subjective well-being, rather than justifying the inclusion of mental health as a mediator. A more logical and robust argument is needed to justify why mental health is the focus of your study.

**Response 2:** We appreciate the reviewer’s insightful comment. In response, we have revised the third paragraph of the introduction to provide a more theoretically grounded and logically cohesive justification for selecting mental health as the mediating variable. Rather than focusing solely on usage patterns or population differences, we now draw on psychological pathway mechanisms and relevant theories such as Socioemotional Selectivity Theory and Life Course Theory. These frameworks emphasize how age-related psychosocial transitions influence emotional regulation and mental adaptation, thereby highlighting mental health as a core mechanism through which internet use may affect subjective well-being among older adults.

We have incorporated this rationale to better align with the mediating focus of our study and added references to support our argument. These changes clarify the theoretical contribution of the mental health pathway and address the concern regarding its conceptual justification.

Manuscript revision location: Introduction, lines 68–86.

**Comment 3:** There is a need to identify gaps in existing studies on the relationship between internet use and well-being to justify the theoretical implications of your research. Introducing such research gaps would help strengthen the introduction and highlight the relevance of this study.

**Response 3:** Thank you for this insightful comment. We agree that identifying the research gaps is crucial to demonstrating the theoretical relevance of our study. In the revised introduction, we have added a paragraph that explicitly highlights the limitations of existing research, particularly the lack of attention to psychological mechanisms linking internet use and subjective well-being among older adults. While prior studies have often focused on direct associations, few have systematically examined how mental health functions as a mediating pathway. By emphasizing this gap, we aim to clarify the theoretical contribution of our study and provide a stronger rationale for exploring mental health as a core mechanism in this relationship.

Manuscript revision location: Introduction, lines 87–98

**Comment 4:** In the first section of the literature review, you wrote: “Despite these insights, existing research has primarily emphasized the outcomes of internet use on well-being, while neglecting the internal psychological processes through which such effects occur. Specifically, the role of psychological states—such as anxiety, loneliness, and goal perception—has received limited attention as potential mediators.” This statement suggests that your selection of these mediators was somewhat arbitrary and lacks theoretical justification. Please explain why you focus on psychological states and, specifically, why you chose anxiety, loneliness, and goal perception as mediators.

**Response 4:** We sincerely appreciate the reviewer’s insightful comment regarding the justification of our chosen mediating variables. In response, we have added a more detailed theoretical explanation to support the inclusion of psychological anxiety, social loneliness, and goal deficiency as mediators in the relationship between internet use and subjective well-being.

Firstly, psychological anxiety is a fundamental emotional response to perceived uncertainty or stress, which is highly relevant in the digital context. According to Pearlin’s stress process model [24], exposure to complex digital information or excessive online stimulation may intensify psychological pressure, especially among older adults with lower digital literacy. Thus, internet use may influence well-being by either alleviating or exacerbating anxiety, depending on usage patterns.

Secondly, social loneliness represents a perceived lack of meaningful social ties. As Weiss [26] proposed in his theory of relational loneliness, older individuals are especially prone to feelings of isolation due to reduced social roles in later life. The internet, while offering communication tools, may not always substitute for authentic offline relationships. Therefore, loneliness serves as a critical pathway through which internet use affects emotional well-being.

Thirdly, goal deficiency is linked to an individual’s sense of direction and purpose in life. Drawing from Self-Determination Theory by Deci and Ryan [25], individuals require autonomy and competence to sustain psychological wellness. When older adults engage in internet use without purposeful engagement—such as passive scrolling—they may experience a diminished sense of personal achievement, contributing to goal deficiency. Conversely, when online activities align with intrinsic motivations, the internet can help strengthen one’s life purpose.

These three constructs are not arbitrarily selected but are grounded in well-established psychological theories. They represent emotional (anxiety), relational (loneliness), and motivational (goal perception) domains of mental health, thus offering a comprehensive framework to understand how digital engagement translates into subjective well-being outcomes. We have incorporated these theoretical foundations into the “Internet Use and Mental Health” and “Mental Health and Subjective Well-Being” sections of the literature review to clarify our rationale.

Manuscript revision location: Literature review, lines 99–164.

**Comment 5:** Throughout the introduction and literature review, you refer to “internet use,” while in the hypotheses section, you focus on “social media use.” These are distinct concepts, and the inconsistency may confuse readers. Please use consistent terminology throughout the manuscript to avoid misunderstandings.

**Response 5:** We sincerely thank the reviewer for pointing out the inconsistency in terminology between “internet use” and “social media use.” We fully acknowledge that these terms refer to distinct concepts, and such inconsistency could lead to confusion regarding the study’s focus and scope.

To address this issue, we have carefully reviewed the entire manuscript and standardized the terminology to consistently use “internet use” throughout, including in the hypotheses section, methods, and discussion. All references to “social media use” have been revised accordingly to ensure conceptual clarity and alignment with our research framework.

We appreciate the reviewer’s careful attention to detail, which has helped improve the coherence and rigor of the manuscript.

Manuscript revision location: Throughout the manuscript, including the Hypotheses section (Lines 178–203).

**Comment 6:** Each hypothesis lacks sufficient elaboration, making the current literature review appear weak. At least three references should be provided to justify each hypothesis. For example, you did not cite any literature to support the hypothesized relationship between internet use, goal deficiency, and subjective well-being. Please include relevant references to strengthen the justification for your hypotheses.

**Response 6:** Thank you very much for this valuable suggestion. We agree that the initial version of the manuscript lacked adequate theoretical elaboration and supporting references for the stated hypotheses, particularly regarding the link between internet use, goal deficiency, and subjective well-being.

In response, we have revised the Research Hypotheses section to include more detailed theoretical justifications for each hypothesis. For each proposed pathway, we have now incorporated at least three relevant, high-quality references to support the rationale, drawing upon recent literature in aging, mental health, and digital behavior. Specifically, for H4, we added references such as Zhang and Zhou (2022), Sum et al. (2008), and Nie et al. (2023), which empirically demonstrate that excessive or unstructured internet use may reduce older adults’ goal clarity and perceived life purpose, thereby negatively impacting subjective well-being.

These modifications not only reinforce the theoretical foundation of our study but also help clarify the conceptual pathways being tested. We hope these improvements will enhance the scholarly rigor and clarity of the manuscript.

Manuscript Revision Location: Research Objectives and Hypotheses, Lines 178–203 (Theoretical justifications and references have been added before each hypothesis).

**Comment 7:** In the introduction and literature review, please explain why your study focuses on older adults. Are there unique patterns of internet use within this population? Does internet use have distinctive impacts on their mental health or well-being? More elaboration on the research context is needed to clarify the rationale behind focusing on older adults.

**Response 7:** We thank the reviewer for this insightful comment. In the revised version of the manuscript, we have strengthened the rationale for focusing on older adults by clarifying the unique characteristics that make this population especially relevant to the research question.

First, we emphasized that older adults exhibit distinct internet use patterns, often using digital platforms for emotionally meaningful goals—such as staying in touch with family, seeking health-related information, and alleviating loneliness—which differ substantially from younger users’ motivations (e.g., entertainment or work).

Second, older adults are psychologically more vulnerable due to age-related life transitions (e.g., retirement, bereavement, health decline), and their subjective well-being tends to be more sensitive to emotional states and social connectivity. Research has shown that this group faces a higher risk of mental health challenges, including loneliness, anxiety, and a reduced sense of purpose, making them an ideal population for studying how internet use may help or hinder well-being through psychological pathways.

Third, given the rapid digital inclusion of older adults in countries like China, this group represents both a policy and scientific priority for understanding how to promote healthy aging in the digital age.

Manuscript revision location: Literature review, lines 58–67.

**Comment 8:** In the “Statistical Analysis” section, you mentioned: “Building on this, mediation effects were tested to explore the mechanisms among internet use, alienation, and mental health. The mediating role of alienation in the relationship between internet use and mental health was examined.” However, “alienation” does not appear in the results. Why is this variable mentioned here but not included in the findings? Additionally, why did you not include a structural equation modeling (SEM) approach to test mediation effects in this section?

**Response 8:** Thank you for pointing this out. We acknowledge that the mention of “alienation” in the original statistical analysis section was an oversight. The term was used in an earlier draft of the manuscript during exploratory discussions of potential mediators but was later removed from the analysis due to insufficient theoretical fit and measurement limitations. We have now deleted the reference to “alienation” in the Statistical Analysis section to maintain consistency with the final reported results.

In response to your second point, we confirm that we did use structural equation modeling (SEM) to test the mediating effects of psychological anxiety, social loneliness, and goal deficiency between internet use and subjective well-being. The analysis was conducted using the lavaan package in R, and the results are presented in Table 4 of the revised manuscript. The significance of indirect effects was assessed using the bias-corrected bootstrap method with 5,000 resamples. We have revised the Statistical Analysis section accordingly to clarify this methodological approach.

Manuscript changes: Statistical Analysis section, lines 275-285.

**Comment 9:** When reporting the correlation analysis in the results, you wrote: “These results indicate that subjective well-being among older adults is influenced by internet use, education, and psychological status. Thus, promoting positive internet engagement, expanding educational access, and strengthening mental health support may be beneficial to the well-being of this population.”
However, as this is a cross-sectional survey, causal relationships between variables cannot be inferred. Please avoid using the term “influence” when reporting correlation results. Additionally, the suggested implications (e.g., promoting positive internet engagement) should be moved to the discussion section, as they do not belong in the results.

**Response 9:** Thank you for your insightful comment. We agree that using the term “influence” in the context of correlation analysis within a cross-sectional study may lead to misinterpretation of causality. We have revised the wording in the Results section to use neutral expressions such as “associated with” and “correlated with,” avoiding any implication of causality. Additionally, we have removed the recommendation-oriented sentences (e.g., “promoting positive internet engagement”) from the Results section and relocated them to the Discussion section, where such implications are more appropriate.

Manuscript changes: Statistical Analysis section, lines 306-323.

**Comment 10:** In the section “The Relationship Between Internet Use and Subjective Well-Being Among Older Adults,” there are inconsistencies in how standard errors (SE) are reported. For some correlations, SE is provided, while for others, it is not. Please ensure consistent reporting of results and clarify the reasons for these discrepancies, if any.

**Response 10:** We sincerely thank the reviewer for pointing out the inconsistency in the reporting of standard errors (SE) in the results section. Upon careful review, we found that this issue stemmed from differences in the types of statistical models reported in different tables. Specifically, SEs were consistently included in the OLS regression results (Table 3), where they are typically presented in parentheses beneath each coefficient. However, in the original SEM output (Table 4), standard errors were inadvertently omitted due to format constraints during initial table construction.

To address this concern, we have revised Table 4 by adding a dedicated “Standard Error” column to accompany all reported path coefficients, z-values, and p-values. This ensures that the presentation is now fully aligned across all analytical sections. We believe this correction enhances the clarity, transparency, and comparability of the findings.

We thank the reviewer again for this valuable suggestion, which helped us improve the rigor and reporting consistency of our statistical results.

Manuscript changes: Statistical Analysis section, lines 374-376.

**Comment 11:** What does “-Cons” mean in Table 3? Please clarify or provide a footnote to explain this abbreviation.

**Response 11:** Thank you for your helpful comment. We acknowledge that the abbreviation “–Cons” in Table 3 may be unclear to readers unfamiliar with statistical conventions. To enhance clarity and readability, we have revised the label and replaced “–Cons” with the more explicit term “Constant” to represent the intercept in the regression models.

Manuscript changes: Statistical Analysis section, lines 361-362.

**Comment 12:** In Table 4, the total effect of internet use on subjective well-being is reported as 0.066. However, this value does not equal the sum of the direct effect (0.059) and the total indirect effect (0.029). Please provide an explanation for this discrepancy.

**Response 12:** We sincerely thank the reviewer for this insightful observation. The noted discrepancy between the total effect (0.066) and the sum of the direct effect (0.059) and the total indirect effect (0.029) in Table 4 is due to the use of standardized path coefficients in structural equation modeling (SEM).

In SEM, standardized coefficients are calculated separately for each path based on the variances of the respective variables involved. Since each path is standardized independently, the sum of individual standardized effects may not perfectly equal the reported total standardized effect. This is a recognized characteristic of SEM using standardized estimates and does not reflect a calculation error nor does it undermine the reliability of the results.

To address this issue and improve clarity, we have added an explanatory footnote under Table 4 in the revised manuscript, stating:

Note: The total effect (0.066) differs slightly from the sum of the direct (0.059) and indirect (0.029) effects because standardized SEM coefficients are estimated separately and may not add up exactly. This does not affect result validity.

We hope this clarification adequately addresses the reviewer’s concern.

Manuscript changes: Statistical Analysis section, lines 377-379.

**Comment 13:** The title of Table 5 reads: “Table 5. Pearson’s correlations among relevant study variables.” However, the table seems to pertain to the model fit of SEM. Additionally, you did not report SRMR and CFI values for the model, which are essential for assessing model fit. Please revise this section and refer to Hu and Bentler (1999) for appropriate model fit criteria: Hu, L., & Bentler, P. M. (1999). Cutoff criteria for fit indexes in covariance structure analysis: Conventional criteria versus new alternatives. Structural Equation Modeling, 6(1), 1–55. https://doi.org/10.1080/10705519909540118

**Response 13:** Thank you for your helpful observation. We acknowledge that the original title of Table 5 may have caused confusion. While the intention was to present model fit indices for the structural equation model (SEM), the title mistakenly referred to “Pearson’s correlations.” To ensure clarity and alignment with the table content, we have revised the title to:

“Table 5. Fit Indices of the Structural Equation Model.”

In addition, we have incorporated key fit indices such as CFI and SRMR, following the recommendations of Hu and Bentler (1999), to strengthen the assessment of model adequacy. These revisions are now reflected in both the title and the table content.

Manuscript changes: Statistical Analysis section, lines 394.

**Comment 14: I**n the discussion section, avoid repeating the findings already reported in the results. Instead, compare your findings with those of existing studies, provide plausible explanations, and suggest implications. Please remove statistical details from the discussion section and focus on strengthening the theoretical implications of your findings.

**Response 14:** We thank the reviewer for this constructive comment. In response, we have carefully revised the Discussion section to remove repetitive statistical descriptions and instead strengthen the theoretical analysis of our findings.

In the previous version, statistical indicators such as β coefficients and p-values were repeated throughout the discussion. These have now been removed to avoid redundancy.

•	For H1, we now explain the result in terms of flow theory, emphasizing the emotional enrichment from meaningful online engagement [57,58].

•	For H2 and H4, we draw on social compensation theory to interpret how internet use may buffer negative emotions like anxiety or restore a sense of purpose [59,15].

•	For H3, we no longer emphasize statistical insignificance. Instead, we provide a theoretical explanation for the limited effect of internet use on alleviating social loneliness, especially among older adults with low digital literacy [60].

•	The paragraph discussing H5 focuses on the integrated mediating role of mental health and positions the findings within broader psychological frameworks.

We hope these changes meet your expectations and enhance the manuscript’s theoretical clarity and academic contribution.

Manuscript changes: Statistical Analysis section, lines 395-427.

**Comment 15:** Please include a discussion of the study’s limitations, such as the measurement of key variables, to clarify the generalizability of your findings. Addressing limitations will enhance the transparency and rigor of your study.

**Response 15:** Thank you for this insightful suggestion. We appreciate the reviewer’s emphasis on transparency and rigor. In response, we have added a dedicated paragraph at the end of the Discussion section to address the limitations of our study. Specifically, we now acknowledge the following: (1) the potential for measurement error due to reliance on self-reported, single-item indicators; (2) the inability to infer causality due to the cross-sectional nature of the CGSS data; (3) limited generalizability, as the sample consists solely of older adults in mainland China; and (4) the possible influence of unmeasured factors such as personality traits or offline social networks on the studied relationships. By explicitly outlining these limitations, we aim to provide a more balanced interpretation of our findings and suggest directions for future research.

Manuscript changes: Statistical Analysis section, lines 428-441.

Once again, we sincerely thank the academic editor and reviewers for your constructive suggestions and valuable guidance. Your insights have played a vital role in improving the manuscript. We hope the revised version has adequately addressed your comments and is now suitable for publication in PLOS ONE.

Should there be any further questions or revisions required, we will be more than willing to cooperate. Thank you again for your time and consideration.

With kind regards,

Chunyun Tan

Corresponding Author (on behalf of all authors)
