# Supplementary material for: The impact of internet use on the subjective well-being of older adults: The mediating role of mental health
Source: PLoS One. 2025 Oct 31;20(10):e0335985. doi: 10.1371/journal.pone.0335985 (PMC12578345; doi:10.1371/journal.pone.0335985)
Supplement: Attachment — Submitted filename: Response_to_Reviewers_auresp_3.docx [file pone.0335985.s004.docx]

**Response to Academic Editor and Reviewers**

**Manuscript Title: *The Impact of Internet Use on the Subjective Well-Being of Older Adults: The Mediating Role of Mental Health***

**Manuscript Number: [PONE-D-24-57555]**

Dear Academic Editor and Reviewers,

We would like to express our sincere gratitude to the academic editor and all reviewers for your thorough evaluation and constructive feedback on our manuscript. We greatly appreciate the time and effort you invested in helping us improve the clarity, conceptual grounding, and methodological rigor of this study.

In response to each comment, we have carefully revised the manuscript and provided point-by-point answers below. For clarity, each reviewer comment is restated, followed by our detailed response and the specific location of the corresponding changes in the revised manuscript.

**Response to Reviewer 3’s Comments:**

**Comments 1:** Citations Needed:

a. The content about the uniqueness of older adults from lines 61 to 67 needs citations:

“Older adults exhibit unique patterns of internet use, often centered around maintaining family contact, acquiring health-related information, or alleviating loneliness. At the same time, they are more vulnerable to mental health challenges due to life course transitions, reduced social networks, and cognitive aging. Compared to younger cohorts, older adults’ subjective well-being is more sensitive to changes in emotional states and social connectedness. These characteristics make them a critical group for examining how internet use influences well-being through psychological mechanisms.”

b. Similarly, “Older adults are especially sensitive to psychological vulnerabilities, as they often experience transitions such as retirement, bereavement, and social role reduction, which may affect their emotional and cognitive well-being.” (Lines 71-73) needs citation.

c. “Despite growing scholarly interest in how internet use affects the subjective well-being of older adults, existing studies have primarily concentrated on its direct effects, with insufficient attention to the underlying psychological mechanisms. In particular, the mediating role of mental health remains underexplored. Although previous research has shown associations between internet use and emotional outcomes, few studies have systematically investigated how specific psychological constructs—such as anxiety, loneliness, or goal-related cognition—function as mediators in this relationship. Addressing this gap is essential for advancing theoretical understanding and informing targeted mental health interventions in digitally connected aging populations.” (Lines 87-95) also needs citation.

d. “For instance, older adults experiencing lower anxiety and loneliness are more likely to benefit from online interactions, while those who lack a sense of life purpose may not derive meaningful satisfaction from internet use.” (Lines 157-159) needs citation.

**Response 1:** We thank the reviewer for the insightful and constructive comments regarding the need for additional citations to support key statements about older adults’ internet use patterns, psychological vulnerabilities, and the mediating role of mental health. We fully agree that providing robust empirical and theoretical evidence will strengthen the credibility and academic rigor of our manuscript.

Accordingly, we have carefully reviewed recent high-quality publications indexed in SSCI/SCI journals and other authoritative sources and have supplemented the relevant sections with appropriate citations. Specifically:

A. For Comment a (Lines 61–67), we have added empirical studies such as Cotten et al. (2013) and Urbaniak et al. (2023), which document older adults’ distinctive internet use purposes (e.g., maintaining family ties, reducing loneliness) and the impact of life-course transitions on social relationships and well-being. We have also included Siedlecki et al. (2014) to highlight the association between social support and subjective well-being across age.
B. For Comment b (Lines 71–73), we have incorporated Li et al. (2022), which provides national longitudinal evidence from China showing that internet use can buffer the negative effects of social isolation on depression and cognitive function among older adults.
C. For Comment c (Lines 87–95), we have cited Chen and Persson (2002), Heo et al. (2015), and Tsai et al. (2025), which examine the relationship between internet use and psychological well-being, and identify technology anxiety, loneliness, and other psychological constructs as potential mediators in technology adoption and well-being outcomes.
D. For Comment d (Lines 157–159), we have included Hofer and Hargittai (2024), which empirically demonstrates that online social engagement is associated with lower levels of depression and anxiety among older adults, supporting our argument that reducing psychological distress can enhance the benefits of internet use.

We believe these revisions provide stronger support for the key arguments by incorporating recent, high-quality scholarly evidence, which we hope addresses the reviewer’s concerns and further strengthens the manuscript’s theoretical and empirical grounding.

Manuscript revision location: lines 81–240.

**Comment 2-a:** Theoretical Arguments:

a. “According to Pearlin’s stress process model [24], exposure to complex digital information or excessive online stimulation may intensify psychological pressure, especially among older adults with lower digital literacy.” However, Pearlin’s stress process model did not suggest the premise that you propose—that online information can be a source of stress. Please check this citation to avoid mis-citation.

In your literature, you also mentioned, “On the other hand, excessive or unregulated internet use may lead to information overload, social comparison, and reduced real-life interaction, which can heighten psychological anxiety and emotional exhaustion [15]. This phenomenon can be interpreted through the Stress Process Model [24], which posits that environmental demands—such as digital complexity—may trigger anxiety unless appropriate coping resources are in place.” (Lines 127-132). I don’t agree with this argument that internet use turns out to be an environmental demand that triggers people’s anxiety. You may consider interpreting the negative impacts of internet use on mental health from other theoretical perspectives.

**Response 2-a:** We sincerely appreciate the reviewer’s insightful comment and fully agree with the concern regarding the misapplication of Pearlin’s Stress Process Model. Upon careful re-examination of the original model, we acknowledge that it primarily addresses the role of stressors, mediators, and outcomes in the context of life stress, but it does not explicitly conceptualize online information exposure or digital complexity as environmental demands. We agree that our earlier framing risked overstating the model’s scope.

In response, we have revised the relevant section to remove the direct attribution to Pearlin’s Stress Process Model when describing the potential negative impacts of internet use. Instead, we now interpret these effects through a framework more directly aligned with technology-related stress research—drawing on the concept of digital stress as operationalized by Fischer et al. (2021), which captures stress perceptions arising from technology-related demands, including information overload, digital communication overload, and exposure to potentially misleading information. This theoretical adjustment ensures that our interpretation is supported by an established framework in the digital era and avoids mis-citation.

Manuscript revision location: Literature review, lines 192–197.

**Comment 2-b:**

b. Additionally, you acknowledge that “internet use may influence well-being by either alleviating or exacerbating anxiety, depending on usage patterns.” Internet usage patterns are important, but this study did not examine them, thus making the rationale for the hypothesis quite weak.

**Response 2-b:** We thank the reviewer for this helpful comment. In the revised manuscript, we have removed the reference to “usage patterns” to avoid implying that this study directly examined such variables. Instead, we have clarified that our analysis focuses on the overall frequency of internet use and its association with mental health outcomes.

**Comment 3-a: Literature Review**

a. In the literature review “Internet Use and Mental Health,” you concluded, “Taken together, these findings suggest that the effects of internet use on mental health are not linear, but mediated by key psychological variables.” However, according to your theoretical arguments, it seems that the relationships between internet use and different aspects of mental health mainly depend on Internet usage patterns, such as excessive or unregulated internet use, and digital engagement that lacks meaningful goal orientation. However, your study did not measure older adults’ usage patterns; instead, you only measured their frequency of internet use. Thus, the current theoretical argument seems not to provide a strong rationale for your research focus and hypothesis.

**Response 3-a:** We sincerely thank the reviewer for the helpful guidance. To better align our theoretical framing with the variables analyzed, we now ground our rationale in Self-Determination Theory, Socioemotional Selectivity Theory, and the life-course/transactional stress–coping perspective, which collectively support treating internet-use frequency as a meaningful exposure for affective and relational processes. We have removed wording that could imply reliance on unmeasured usage patterns and added frequency-focused empirical evidence (Heo et al., 2015; Cotten et al., 2013; Li et al., 2022). The revised paragraph clarifies that our mediation analysis examines how anxiety, social loneliness, and goal deficiency transmit the association from use frequency to subjective well-being in later life. We appreciate this suggestion, which has helped us tighten the logic and maintain a cautious, association-based interpretation of results.

Manuscript revision location: Literature Review, lines 198–230.

**Comment 3-b:**

b. In your response to Comment 4, you mentioned why you chose anxiety, loneliness, and goal perception as mediators: “These three constructs are not arbitrarily selected but are grounded in well-established psychological theories. They represent emotional (anxiety), relational (loneliness), and motivational (goal perception) domains of mental health, thus offering a comprehensive framework to understand how digital engagement translates into subjective well-being outcomes.” This argument looks reasonable, but why don’t you justify it in the introduction and literature review?

**Response 3-b:** Thank you for the helpful suggestion. We now justify our choice of the three mediators in the Literature Review by adding concise, theory-anchored sentences that map each mediator to a relevant framework. For example, we state:

•Anxiety: “From a stress–coping perspective, higher overall exposure to digital demands may be appraised as strain when coping resources are limited; therefore, we treat anxiety as a proximal affective pathway linking internet-use frequency to well-being [31,32].”

•Loneliness: “At the same time, age-related changes can erode social roles and network structure; as Weiss’s relational loneliness framework highlights, older adults are therefore especially susceptible to isolation, underscoring the salience of the loneliness pathway in later life [33].”

•Goal deficiency: “Grounded in Self-Determination Theory, psychological well-being depends on a sustained sense of purpose and competence; accordingly, overall internet-use frequency can shape affective and relational states that support—or undermine—these needs [34,35].”

These additions clarify why loneliness), goal deficiency, and anxiety are appropriate mediators and align the argument with our measured exposure (internet-use frequency).

Manuscript revision location: Literature Review, lines 196–203.

**Comment 4-a: Research Objective & Hypotheses:**

a. The research objective, “The aim of this study is to examine the relationships among internet use, mental health, and subjective well-being (SWB) in older adults, with a particular focus on the mediating role of mental health. Although internet use among older adults is increasingly prevalent, the psychological mechanisms linking internet use to well-being remain insufficiently understood. Drawing on nationally representative data from the China General Social Survey (CGSS), this study investigates how internet use influences SWB through three specific psychological dimensions: psychological anxiety, social loneliness, and goal deficiency,” should be moved up to the end of the introduction instead of being positioned behind the literature review.

**Response 4-a:** Thank you for the valuable suggestion. We have repositioned the research objective paragraph to the end of the Introduction to neatly close the motivation and research gap, enhance narrative coherence, and provide a clear bridge to the subsequent hypotheses and analytic framework.

Manuscript revision location: Introduction, lines 141–147.

**Comment 4-b, c:**

b. The hypotheses section should also be inserted into the literature review when you state the relationship between key variables. The current formatting is redundant and repetitive.

c.H5 is actually the composition of H2-4. Why bother to include it? Additionally, the relationships between internet use and the three mental health dimensions are missing in the current hypotheses.

An alternative way is to propose hypotheses on the relationship between internet use and the three mental health dimensions and then keep the current H5 (i.e., the mediation effect of the three mental health dimensions in the relationship between internet use and subjective well-being). This can also help readers understand the direction and valence of each path.

There are many tutorials about how to propose direct and mediating hypotheses in research. You may consider referring to them.

**Response 4-b, c:** Thank you very much for the thoughtful guidance. We have carefully revised the manuscript to reduce redundancy, tighten theory–evidence–hypothesis linkages, and improve readability:

1.Embedding within the Literature Review. To avoid a stand-alone, repetitive “Hypotheses” section and to keep a clear theory→evidence→hypothesis flow, each hypothesis is now placed at the end of its corresponding Literature Review subsection: H1 after Internet Use & Subjective Well-Being; H2–H4 after Internet Use & Mental Health; H5 after Mental Health & Subjective Well-Being. We also added brief transition sentences immediately before each hypothesis to lead in the proposition smoothly.

2. Direct associations added. In line with your suggestion, we made the previously implicit direct links explicit:

•H2: Internet use is negatively associated with psychological anxiety.

•H3: Internet use is negatively associated with social loneliness.

•H4: Internet use is negatively associated with goal deficiency.

3. Mediation clarified. We retain H5 as a concise test of the overall indirect effect via the three mental-health dimensions (parallel mediation).

Manuscript revision location: Literature review, lines 176,238,281.

**Comment 5-a:** **Measurement**

a. “Variable manipulation” is often used in experiments when you manipulate a variable through stimuli. Please change it to “variable” or just remove it.

**Response 5-a:** Thank you for the helpful clarification. We have removed the term “variable manipulation”—which is inappropriate for an observational study—and replaced it with “variables”

Manuscript revision location: Measure, lines 403.

**Comment 5-b:**

b. Your conceptualization of the three dimensions of mental health and justification should be incorporated into the literature review instead of being presented in the operationalization of measurement.

**Response 5-b:** We are grateful for this constructive guidance. We have relocated the conceptualization and theoretical justification for the three mental-health dimensions (psychological anxiety, social loneliness, goal deficiency) from the Measures section to the Literature Review. Specifically, brief theory-anchored statements now appear in the “Internet Use and Mental Health” and “Mental Health and Subjective Well-Being” subsections to justify why these constructs are relevant mediators. The Measures section now only reports operational details (CGSS items, response scales, coding, and summary statistics), with a cross-reference to the Literature Review for conceptual grounding.

Manuscript revision location: Measure, lines 421–429.

**Comment 5-c:**

c.In measurement, the symbol of mean (M) and standard deviation (SD) for each variable should be italicized. Additionally, why did you not include the values of the mean and standard deviation for other variables, such as SWB, internet use, and control variables? Please go through your manuscript closely to ensure that all statistics are reported consistently.

**Response 5-c:** Thank you for the suggestion. To ensure consistency and avoid duplication, we now report all means and standard deviations exclusively in the Results → “Descriptive statistics” section. The Measures subsection only defines constructs and items (no numerical summaries). In addition, all statistical symbols (e.g., M, SD, r, p) are now italicized throughout the manuscript.

**Comment 6: Statistical Analysis:**

Please indicate how you evaluate the model fit of the SEM model, specifically, which indicators you use and which cut-off values you used for benchmarking, including citations.

**Response 6:** We are grateful for the reviewer’s thoughtful guidance. Following commonly recommended SEM cutoffs and Kline (2016), we use the suggested thresholds χ²/df < 3, RMSEA ≤ 0.06, CFI ≥ 0.95, SRMR ≤ 0.08, and GFI/AGFI/NFI/TLI/IFI ≥ 0.90. The “Acceptable values” row in Table 5 lists these benchmarks for evaluation. We sincerely appreciate the reviewer’s suggestions, which have improved the clarity and rigor of our reporting.

Manuscript revision location: Results, lines 638–641.

**Comment 7-a:** **Results**

a. In descriptive statistics, “Regarding place of residence, 94.3% of the participants lived in rural areas, while only 5.7% were from urban areas.” Is this true? Why does the majority of older adults in the sample come from rural areas instead of urban areas, which is quite counterintuitive? Isn’t internet access among older adults in urban areas higher than those in rural areas? Given this sample characteristic, the findings of this study have limitations in generalizability. Please acknowledge this in the limitations.

**Response 7-a:** Thank you for raising this important point. The China General Social Survey (CGSS) is a nationally representative, multistage, stratified probability sample covering both urban and rural areas across provinces. In CGSS, place of residence is classified primarily by hukou (household registration) status. Among older cohorts in China, the historical hukou structure and cohort-specific urbanization patterns mean that a higher proportion of individuals aged 60+ hold rural hukou relative to younger cohorts. When restricting the CGSS to older adults, this yields a rural-heavy subsample, which explains the high rural share reported in our descriptive statistics.

To address your concern, we have flagged this explicitly in Discussion → Limitations, noting that the rural-heavy composition may constrain generalizability to urban older adults and that, where appropriate, applying CGSS population weights can better align estimates with national distributions.

Manuscript revision location: Discussion, lines 733–756.

**Comment 7-b, c, d:**

b. The symbol of correlation and p-value should also be italicized.

c.When reporting the results of “Model 3: Inclusion of Psychological Anxiety,” “of birth (B = –0.013, p < 0.05) and education (B = 0.092, p < 0.05) remain significant; gender and residence remain insignificant.” “Insignificant” is the wrong expression; please replace it with “non-significant.”

d.The font of “Standard Error” in Table 4 is different from other values. Please keep all fonts of values consistent.

**Response 7-b, c, d:** Thank you for these careful observations. We have made the following corrections:

1. Italics: in the main text, the correlation symbol (e.g., r) and p are now consistently italicized.

2. Wording: all occurrences of “insignificant” have been replaced with “non-significant,” including in the description of Model 3.

3. Formatting: the font for “Standard Error” in Table 4 has been unified to match the other entries; we also checked all tables for consistency.

**Comment 7-e:** When reporting the results in “Mediating Effect Analysis,” you should always address the hypotheses you proposed in the literature review to show readers which ones are supported and which ones are rejected.

**Response 7-e:** Thank you for the helpful guidance. We have revised the Mediating Effect Analysis subsection to explicitly tie the results to each hypothesis (H1–H5) and to state which are supported or not, with cross-references to Tables 4–5. The text now reads, in essence: “internet use is associated with lower psychological anxiety, social loneliness, and goal deficiency (supporting H2–H4); the direct effect on SWB is not significant (thus H1 not supported). The total indirect effect is significant (supporting H5); indirect effects operate chiefly via psychological anxiety and goal deficiency, whereas the loneliness pathway is only marginal (p = 0.072).”

Manuscript revision location: Results, lines 617–637.

**Comment 8: Discussion:**

a. You explained the positive association between internet and subjective well-being. However, the results of the Stepwise OLS Regression Analysis and SEM both suggest these two variables have almost non-significant correlation. Given this, how can you still draw such a conclusion? Doesn’t the non-significant association between internet use and subjective well-being suggest that internet use itself cannot predict subjective well-being? Instead, usage patterns (i.e., how they use the internet) matter in this process.

**Response 8:** Thank you for this careful observation. We agree that the direct association between internet use and subjective well-being (SWB) is non-significant in both the stepwise OLS and the SEM. In light of this, we have removed any language implying a positive direct effect and have reframed the Discussion to characterize the pattern as an indirect-only (mediated) relationship. Specifically, the revised text states that internet use relates to SWB primarily through mental-health pathways, with psychological anxiety (strongest) and goal deficiency (modest) providing significant indirect routes, and the loneliness pathway not reaching significance. Accordingly, we now note that H1 (direct effect) is not supported, whereas the paths specified in H2–H4 are supported, and the overall mediation (H5) is supported.

To avoid implying analyses we did not conduct, we also removed references to “usage patterns” as explanatory mechanisms in the Discussion and now mention them only as future research directions. Practical implications have been aligned with the mediated pattern (e.g., anxiety-reduction supports; goal-oriented digital activities), rather than with a direct association.

We appreciate the reviewer’s guidance, which helped us present a more precise interpretation consistent with the evidence.

Manuscript revision location: Discussion, lines 643–718.

**Comment 9:** What is missing in the current discussion is the practical implications of the findings. How do your findings about the relationships between internet use, mental health, and subjective well-being provide insights for older adults and institutions to improve the well-being of this vulnerable population? Additionally, how can the findings be generalized to other contexts where older adults may have different levels of internet literacy or mental health issues?

**Response 9:** We are grateful for this thoughtful comment. In response, we have added a succinct paragraph to the Discussion that distills the practical implications of our findings: because internet use relates to SWB chiefly via reduced anxiety and stronger purpose/competence, programs for older adults should complement access with light, reassuring supports (to lower digital anxiety), goal-oriented activities, and an emphasis on relationship quality rather than contact volume. We also summarize generalizability: adapt delivery to differing digital-literacy and mental-health profiles, use simple/low-tech options where appropriate, and tailor to local contexts.

Manuscript revision location: Discussion, lines 719–732.

Once again, we sincerely thank the academic editor and reviewers for your constructive suggestions and valuable guidance. Your insights have played a vital role in improving the manuscript. We hope the revised version has adequately addressed your comments and is now suitable for publication in PLOS ONE.

Should there be any further questions or revisions required, we will be more than willing to cooperate. Thank you again for your time and consideration.

With kind regards,

Chunyun Tan

Corresponding Author (on behalf of all authors)
