# Supplementary material for: The impact of internet use on the subjective well-being of older adults: The mediating role of mental health
Source: PLoS One. 2025 Oct 31;20(10):e0335985. doi: 10.1371/journal.pone.0335985 (PMC12578345; doi:10.1371/journal.pone.0335985)
Supplement: Attachment — Submitted filename: Response_to_Reviewers_auresp_4.docx [file pone.0335985.s005.docx]

**Response to Academic Editor and Reviewers**

**Manuscript Title: *The Impact of Internet Use on the Subjective Well-Being of Older Adults: The Mediating Role of Mental Health***

**Manuscript Number: [PONE-D-24-57555]**

Dear Academic Editor and Reviewers,

We would like to express our sincere gratitude to the Academic Editor and the Reviewers for their thorough evaluation and constructive feedback on our manuscript. We greatly appreciate the time and effort they devoted to improving the clarity, conceptual grounding, and methodological rigor of this study.

In response to each comment, we have carefully revised the manuscript and prepared point-by-point replies. For clarity, each reviewer comment is restated, followed by our detailed response and the specific location of the corresponding changes in the revised manuscript.

We sincerely hope that the revised version meets your expectations and is now suitable for consideration of publication in *PLOS ONE*. Once again, we thank you for your valuable time and effort.

**Response to Academic Editor’s Comments：**

**Comments 1:** Please revise your manuscript carefully in accordance with the reviewers’ comments. In addition, I encourage you to give close attention to the overall presentation of the paper. Specifically, please proofread thoroughly to ensure consistency in grammar, spelling, and academic style. Furthermore, double-check all references and citations for accuracy, completeness, and compliance with the journal’s formatting requirements. It is also important to ensure that the language and expressions used in the manuscript accurately reflect the nature of your research, which is based on a cross-sectional survey. In addition, please ensure that terminology is used consistently throughout the manuscript. Finally, review the formatting details—such as headings, subheadings, and spacing—to enhance clarity and readability.

**Response 1:** We sincerely thank the Academic Editor for these detailed comments regarding the overall presentation of the manuscript. In revising the paper, we took the following steps:

1.	Proofreading and consistency:

The entire manuscript was carefully proofread to ensure consistency in grammar, spelling, and academic style. Key terminology (e.g., subjective well-being, internet use, cross-sectional survey) was reviewed and standardized throughout the text.

2.	Clarification of study design:

To accurately reflect the cross-sectional nature of our research, we revised the wording in both the Abstract and Methods sections. Specifically, terms that could imply causality (e.g., “effect,” “impact”) were replaced with more precise expressions such as “association” and “relationship.”

3.	References and citations:

All references were checked one by one for accuracy, completeness, and compliance with the PLOS ONE reference style, which follows the International Committee of Medical Journal Editors (ICMJE) recommendations. Journal abbreviations were verified using the NCBI database. DOI numbers were added where available, and the formatting was aligned with the official guidelines (see: http://journals.plos.org/plosone/s/submission-guidelines#loc-references).

4.	Formatting details:

Headings, subheadings, and spacing were reviewed and standardized across the manuscript. Tables and figure captions were reformatted to ensure clarity, consistency, and adherence to journal standards.

5.	Language refinement:

Expressions in the Abstract, Introduction, and Discussion were revised to improve clarity and academic tone, ensuring that the language accurately conveys the findings and contributions of the study.

We believe these revisions have improved the overall quality and presentation of the manuscript and respectfully submit the revised version for your consideration.

**Response to Reviewer 3’s Comments:**

**Comments 1:** Hypothesis 1 (H1) — "Internet use is positively associated with subjective well-being among older adults" — should be positioned prior to the paragraph beginning with “Despite these insights, existing research has primarily emphasized the outcomes of internet use on well-being, while neglecting the internal psychological processes through which such effects occur.” This paragraph emphasizes the mediating effect of mental health. Additionally, before proposing H1, please provide justification for the positive correlation between internet use and subjective well-being based on prior research.

**Response 1:** We sincerely thank the reviewer for this helpful suggestion. In response, we have repositioned Hypothesis 1 so that it now appears before the paragraph beginning with “*Despite these insights…*”, thereby ensuring that the logical flow proceeds from the direct association between internet use and subjective well-being to the mediating role of mental health.

In addition, we have strengthened the justification for H1 by incorporating recent empirical studies. Specifically, a nationally representative survey from China demonstrated that internet use frequency, the size of online social networks, and digital proficiency were each positively associated with subjective well-being among middle-aged and older adults [26]. Another study focusing on older adults’ online activities reported that communicative uses of the internet had stronger positive effects on eudaimonic well-being than passive or purely informational uses [27]. Comparative research conducted in Finland and Sweden further showed that socially oriented and active internet use was positively related to subjective well-being and psychological health among older populations [28].

The revised paragraph now concludes with the sentence “*Collectively, these studies provide a solid basis for the following hypothesis:*”, which more clearly demonstrates the empirical grounding of H1 and provides a smoother transition from the literature review to hypothesis development.

Manuscript revision location: Literature review, lines 127–138.

**Comments 2:** In the literature review section titled “Internet Use and Mental Health”, after the sentence “The relationship between internet use and mental health among older adults is multifaceted,” please include a definition of mental health as used in your study, specifically outlining its three dimensions.

**Response 2:** We are grateful to the reviewer for this insightful comment. We agree that it is essential to provide a clear definition of mental health as operationalized in our study, especially since it directly underpins the mediating variables tested in our model. Following the reviewer’s advice, we have revised the literature review section accordingly.

Specifically, immediately after the sentence “The relationship between internet use and mental health among older adults is multifaceted,” we have added the following sentence: “In this study, mental health is conceptualized as a multidimensional construct encompassing three aspects: psychological anxiety, social loneliness, and goal deficiency. These dimensions reflect emotional distress, perceived social disconnection, and a diminished sense of purpose in later life.”

This addition makes the scope of “mental health” more explicit, reduces possible ambiguity, and ensures better alignment between the conceptual framework, the empirical measures, and the hypotheses (H2–H4) presented later in the paper. We believe this change strengthens the clarity and coherence of the literature review.

Manuscript revision location: Literature review, lines 148–152.

**Comments 3:** For Hypothesis 5 (H5) — “Internet use affects subjective well-being indirectly through mental health” — please replace “affects” with “is associated with” to avoid implying causality, as your study is based on a cross-sectional survey design.

**Response 3:** We thank the reviewer for this important clarification. We fully agree that causal expressions should be avoided given the cross-sectional design of our study. Following the reviewer’s suggestion, we have carefully revised Hypothesis 5 and all related descriptions throughout the manuscript.

Specifically, the original statement “H5: Internet use affects subjective well-being indirectly through mental health” has been modified to “H5: Internet use is associated with subjective well-being indirectly through mental health.” Corresponding expressions in the results and discussion sections have also been adjusted. For instance:

•	In the mediation results, “indicating that internet use influences well-being primarily through mental-health pathways” has been revised to “indicating that internet use is associated with well-being primarily through mental-health pathways.”

•	In the concluding synthesis, “mental-health variables collectively mediate the association between internet use and SWB” has been retained, but causal wording has been avoided by rephrasing the internal pathway as “digital engagement is associated with well-being largely through its links with lower anxiety and stronger goal-related motivation.”

Manuscript revision location: Literature review, line 231.

**Comments 4:** Consider renaming the title of Table 1 to: “Descriptive Statistics of Key Variables” for clarity.

**Response 4:** We appreciate the reviewer’s helpful suggestion. We have revised the title of Table 1 from “Basic variable description statistics table” to “Descriptive Statistics of Key Variables” to improve clarity and better align with academic writing conventions.

Manuscript revision location: Results, line 325.

**Comments 5:** When reporting the Pearson correlation coefficients among the key study variables, you may report the non-significant results briefly and refer readers to Table 2. Since most of the correlations are significant, this approach will help reduce redundancy in the text.

**Response 5:** We thank the reviewer for this constructive suggestion. We agree that presenting all correlation coefficients in detail within the text created unnecessary redundancy with Table 2. Following the reviewer’s advice, we have revised the correlation analysis section to provide a more concise description.

In the revised version, we highlight only the key significant associations by reporting representative coefficients (e.g., the positive correlation between internet use and subjective well-being, r = .10, p < .01, and the strongest negative correlation between psychological anxiety and subjective well-being, r = –.27, p < .01). Other significant correlations are summarized narratively, while non-significant correlations are briefly noted and referred to Table 2. This adjustment reduces redundancy, improves readability, and ensures that Table 2 remains the main reference for detailed coefficients.

Manuscript revision location: Results, lines 328–338.

**Comments 6:** Please move the results related to H1 — “By contrast, the direct effect of internet use on subjective well-being is not statistically significant (β = 0.059, p = 0.082), so H1 is not supported” — before the results for H2–H4 to maintain the logical flow of hypothesis testing.

**Response 6:** We sincerely thank the reviewer for pointing out this issue with the presentation order. We agree that the results for H1 should logically precede those for H2–H4 to ensure consistency with the sequence of hypothesis testing. Accordingly, we have revised the Results section so that the finding regarding H1—the non-significant direct effect of internet use on subjective well-being (β = 0.059, p = 0.082)—is now presented before the results for H2–H4. This adjustment improves the logical flow of the results while leaving the statistical findings unchanged.

Manuscript revision location: Results, lines 420–429.

**Comments 7:** In your discussion section, please elaborate more clearly on how the findings of this study extend, challenge, or align with existing research. This will help to better justify and highlight your theoretical contributions.

**Response 7:** We sincerely thank the reviewer for this constructive suggestion. In response, we have carefully revised the Discussion section (lines 81–240) to articulate more explicitly how our findings extend, align with, and challenge existing research, thereby highlighting the theoretical contributions of the study.

1.	Extending prior work: By incorporating goal deficiency as an additional mediator alongside psychological anxiety and social loneliness, our study broadens the explanatory framework of how digital engagement influences well-being in later life. This extends previous studies that predominantly examined anxiety or loneliness in isolation.

2.	Aligning with existing evidence: We explicitly situate our results within the “indirect-only” mediation model, emphasizing that the benefits of internet use for older adults’ well-being arise mainly through psychological or social processes rather than direct associations [50,51].

3.	Challenging common assumptions: We note that H1 was not supported, as the direct link between internet use and subjective well-being was non-significant. This challenges the conventional expectation of a straightforward positive relationship and underscores the necessity of considering intermediary psychological mechanisms.

Furthermore, we integrate our findings with recent stratification research, which demonstrates that the impact of internet use on well-being is heterogeneous across subjective social class and is largely transmitted through psychological pathways [54]. Collectively, these revisions allow the Discussion to more clearly demonstrate that digital participation enhances older adults’ well-being primarily by reinforcing psychological resources and sense of purpose, rather than exerting direct effects.

We are grateful for this suggestion, which has helped us refine the discussion and present our theoretical contributions in a clearer and more coherent manner.

Manuscript revision location: Discussion, lines 451–516.

Once again, we would like to express our sincere gratitude to the academic editor and reviewers for your constructive suggestions and valuable guidance. Your insights have been instrumental in improving the quality, clarity, and rigor of this manuscript. We hope that the revised version has adequately addressed all the comments and is now suitable for consideration for publication in *PLOS ONE*. Should there be any further questions or revisions required, we will be more than willing to cooperate. Thank you again for your time and kind consideration.

With kind regards,

Chunyun Tan

Corresponding Author (on behalf of all authors)
